# Development of a neural network model for predicting glucose levels in a surgical critical care setting

**DOI:** 10.1186/1754-9493-4-15

**Published:** 2010-09-09

**Authors:** Scott M Pappada, Marilyn J Borst, Brent D Cameron, Raymond E Bourey, Jason D Lather, Desmond Shipp, Antonio Chiricolo, Thomas J Papadimos

**Affiliations:** 1 University of Toledo, Department of Bioengineering, Toledo, Ohio, USA; 2 University of Toledo Medical Center, Toledo, Ohio, USA; 3 University of Toledo, Center for Diabetes and Endocrine Research, Toledo, Ohio, USA; 4 The Ohio State University Medical Center, Columbus, Ohio, USA

## Abstract

Development of neural network models for the prediction of glucose levels in critically ill patients through the application of continuous glucose monitoring may provide enhanced patient outcomes. Here we demonstrate the utilization of a predictive model in real-time bedside monitoring. Such modeling may provide intelligent/directed therapy recommendations, guidance, and ultimately automation, in the near future as a means of providing optimal patient safety and care in the provision of insulin drips to prevent hyperglycemia and hypoglycemia.

## Background

Following severe trauma, approximately 25% of patients experience hyperglycemia [1]. Sustained hyperglycemia increases mortality and increases care needs [2-4]. However, lowering glucose levels after severe trauma may decrease mortality, days of ventilation, incidence of infection, and length of stay in an intensive care unit (ICU) and in the hospital [2-5]. Aggressive therapy to maintain glucose levels below 150 mg/dl improves outcomes [3]. Glucose levels exceeding 200 mg/dl in severely injured patients have been correlated to an increase in mortality [2]. In addition to trauma patients, cardiothoracic surgical patients also experience lack of glycemic control during all phases of the perioperative period. Persistently elevated glucose values in this patient base have also been linked to adverse outcomes and increases in mortality [6-8].

The standard method for management of glycemic control in critical care patients is adjustment of a variable infusion of insulin on the basis of discrete point of care (POC) blood glucose monitoring via handheld glucose meters [9]. This POC monitoring is completed every 1-4 hours throughout a patient's length of stay in the ICU. Based on POC glucose values, insulin is infused intravenously to maintain a normal glycemic state. This practice is limited as POC monitoring only provides glucose values when measurements are obtained every 1-4 hours. Patients may be hyperglycemic or hypoglycemic between POC results. Recent advances in technology include the development of real-time continuous glucose monitoring (CGM) devices which report measurements of interstitial glucose concentration every few minutes. A recent investigation studied the impact of utilizing real-time CGM in the critical care setting [10]. The study concluded that utilization of CGM did not correlate to a direct benefit in patient outcome. However, utilization of CGM did correlate with improvement of glycemic control in patients with high sequential organ failure (SOFA) scores, and increased the ability of caregivers to mitigate occurrences of hypoglycemia. It is important to note that CGM devices were utilized only for documentation of glucose values and were not used in a predictive capacity. Utilization of CGM devices in combination with predictive models for glucose may provide clinicians with a means for enhancement of glycemic control and patient outcome. Given the limitations of POC monitoring there is a need to develop technologies to allow critical care providers access to predicted glucose values thereby allowing optimization of glycemic control. The prediction of glucose in outpatients with insulin dependent diabetes via a neural network modeling approach has been previously demonstrated [11]. This report is an extension of the previous application, and involves the development and optimization of neural network models for real-time prediction of glucose in critical care patients. Such networks have the ability to quantify the effect of various factors on a desired predicted variable. Neural network modeling is therefore well suited in such a venue, where various factors such as, but not limited to, medications, vital signs, nutritional intake, and ventilation data are routinely collected in a controlled setting. Construction of a neural network model for prediction of glucose in the critical care setting requires a large dataset for model training and development. The utilization of CGM provides a significant and suitable source of glucose data for neural network model development. Such a dataset is superior to a dataset containing only discrete POC values. Furthermore, this allows for assessment of trends in glucose which cannot be distinguished in POC glucose monitoring results.

## Methods

### Patient Data Acquisition and Development of Patient Specific and General Neural Network Models for Prediction of Glucose in the Critical Care Setting

After institutional review board approval a patient specific neural network model was developed/trained using 243.6 hours (2,923 data points) of continuous glucose monitoring (CGM) (Medtronic Diabetes, CGMS Ipro^®^) and concurrent medical records data from a 38 year old trauma patient (who had an intensive care stay of 16 days). The model was configured for prediction of glucose by implementing a prediction horizon of 75 minutes. Additionally, a general feed forward neural network was developed/trained using 515.7 hours (6,188 data points) of CGM and medical records data from 5 critical care patients and configured with the same 75 minute prediction horizon.

### Neural Network Model Design and Training

**Figure 1:**
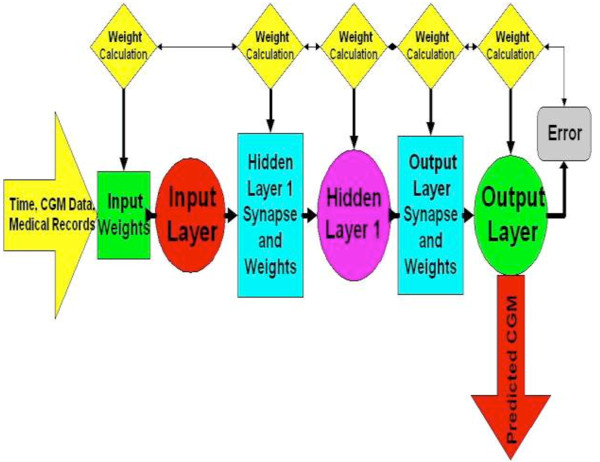
**Neural network architecture and data flow**.

The neural network models were trained via the backpropagation training algorithm. In this training modality, model error is calculated, and model weights for minimization of this error can be determined. The neural network models were designed with a three layer design. The input layer of the neural network was configured to utilize time, CGM data, and electronic medical records that included point of care glucose test times and results, insulin delivery type (intravenous drip, or subcutaneous sliding scale), and units of insulin delivered as input variables for prediction. The second or hidden layer of the neural network was designed to limit the range of neural network model inputs to between -1 and 1. This enables data within the neural network to be more easily processed and for trends in data to be identified more effectively. Figure 1 includes the neural network model architecture and dataflow in generation of model predictions.

### Neural Network Model Performance Analysis and Validation

A computer program implementing the neural network model for real-time prediction of glucose was developed. Data from the trauma patient not utilized for initial model development/training (containing 40.3 hours [484 data points] of CGM and medical records) was used to test the performance of the patient specific and general neural network models. Clarke Error Grid Analysis (CEGA) was completed to assess clinical acceptability of real-time predictions. Overall error (mean absolute difference percent [MAD%]) was calculated for real-time predictions.

CEGA was established in 1987 and was originally utilized to assess patient estimates of blood glucose compared to those obtained using a "gold-standard" reference glucose meter [12]. The accuracy of current CGM technologies is also assessed via utilization of CEGA to compare CGM performance to that of blood glucose meters. Region A contains predicted values within 20% of the reference concentration and Region B contains predictions outside 20%. However, Region B predicted values would not lead to inappropriate treatment. Regions A and B therefore contain predicted values which can be classified as *clinically acceptable*. Region C contains points that lead to unnecessary treatments, and Region D contains points indicating a potentially dangerous failure to detect hypoglycemia. Region E contains predicted values that would confuse treatment of hypoglycemia for hyperglycemia and vice-versa. A successful predictive model and system would thus need a majority of predicted CGM values to fall with regions A and B in the Clarke Error Grid. If a majority of predicted values fall within regions A and B of the Clark Error Grid, any therapeutic interventions made using the NNM predictive results would not lead to any adverse or unwanted glycemic excursions.

### Analysis of the Clinical Applicability and Usefulness of CGM and Neural Network Model for Prediction of Glucose in the Trauma Patient

In addition to performance analysis of the neural network model, the utility of CGM in the trauma patient was analyzed. This was accomplished by determining the percentage of hypoglycemic (≤70 mg/dl) and hyperglycemic (≥150 mg/dl) events that were detected by CGM and not by conventional POC glucose monitoring. In this analysis we locate hypoglycemic and hyperglycemic CGM values and search for POC monitoring values within a defined time window. A time window of 60 minutes is defined as 30 minutes before and 30 minutes after the detected extreme. If there is a POC value within the time window it is defined as a successful detection of hypoglycemia/hyperglycemia via POC monitoring. Table 1 demonstrates the usefulness of CGM in detecting hypoglycemic and hyperglycemic glucose values which are not detected via conventional POC monitoring. This table summarizes the percentage of hypoglycemic/hyperglycemic CGM values detected via POC monitoring.

**Table 1:** Percentages of hyper-and hypoglycemia detected by point-of-care testing

Glucose	% Detected	% Detected at	% Detected at
Range	at 40 min	60 min	80 min
Hyper	51.4	74.0	96.9

Hypo	61.3	91.9	100.0

min = minutes; % = percentage; hyper = hyperglycemia; hypo = hypoglycemia

## Results

Throughout the patient's length of stay in the ICU, CGM detected a total of 642 hyperglycemic and 124 hypoglycemic occurrences. The patient was on an insulin drip throughout their length of stay in intensive care and POC glucose monitoring was completed intensively. Table 1 indicates that the prediction horizon of 75 minutes implemented in the NNMs is likely ideal for this patient population and will provide insight where POC glucose values are not obtained.

**Figure 2:**
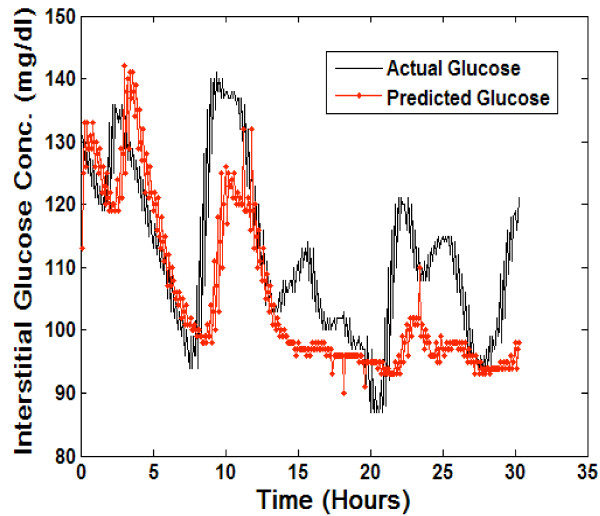
**Real-time predictions generated using patient specific model**. conc. = concentration; mg = milligrams; dl = deciliter.

**Figure 3:**
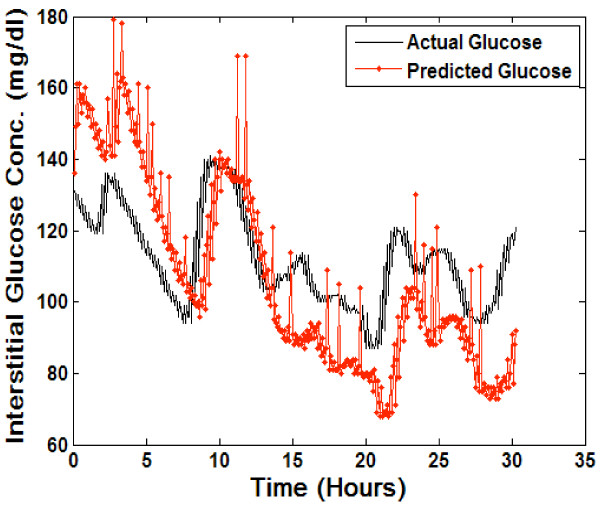
**Real-time predictions generated using general model**. conc. = concentration; mg = milligrams; dl = deciliter.

Figure 2 includes the real-time predictions on the test dataset using the patient specific neural network model. Due to the large dataset of 7,260 predicted glucose values (i.e. 15 CGM values predicted for every CGM value in the test dataset) the data was re-sampled to demonstrate predictive accuracy in Figures 2 and 3. Re-sampling involved plotting every 20th predicted CGM value and corresponding actual glucose value in the predictive dataset. The overall error (MAD%) of the predictions generated using the patient specific model was calculated as 7.9%.

Figure 3 includes the real-time predictions on the test dataset using the general neural network model. The overall error (MAD%) of the predictions generated using the general model was calculated as 15.9%. The patient specific model therefore generates more accurate predictions with a decrease in overall error of 8.0%.

**Figure 4:**
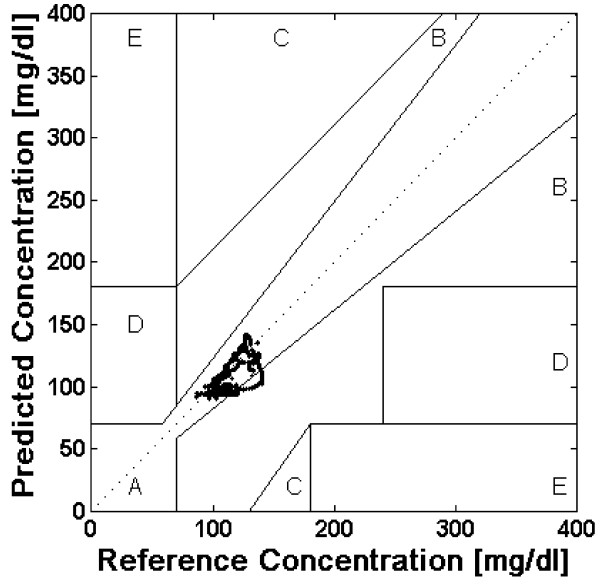
**Clark error grid of predictions generated by patient specific model**. mg = milligrams; dl = deciliter.

**Figure 5:**
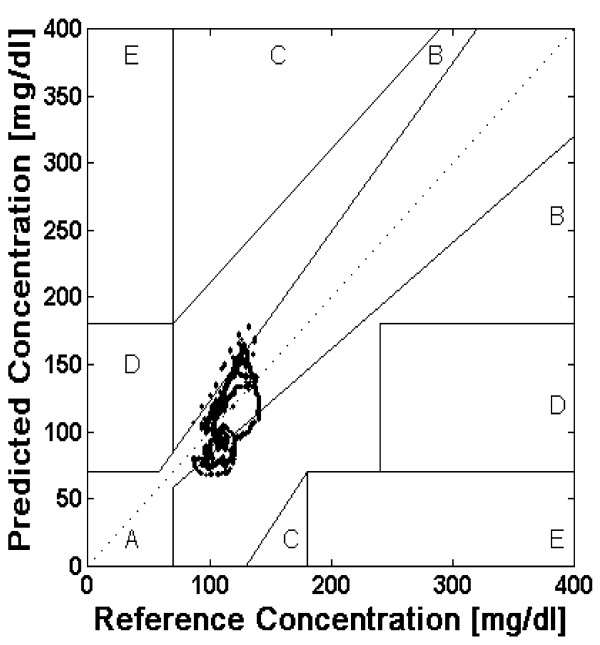
**Clark error grid of predictions by general model**. mg = milligrams; dl = deciliter.

Figure 4 is the Clarke Error Grid showing the real-time predictions generated via the patient specific neural network model. CEGA revealed that 95.1% of the predictions fell within region A of the error grid and 4.9% fell within region B of the error grid. Figure 5 is the Clarke Error Grid showing the real-time predictions generated via the general neural network model. CEGA revealed that 69.8% of the predictions fell within region A of the error grid and 30.2% fell within region B of the error grid. In both instances 100% of the predicted CGM values could be considered *clinically acceptable *with no predicted values falling within regions C, D, or E of the error grid. CEGA also revealed that the patient specific model generated predictions with a high degree of accuracy as 95.1% of the values fell within region A of the error grid and had values within 20% of the reference glucose concentration.

## Discussion

The real-time application of a neural network model for glycemic prediction in critical care patients will provide significant insight to glycemic excursions at points in time where POC glucose values are not obtained. The ability to predict glucose concentration during these time domains would provide caregivers a means of modifying insulin dosages and therapy for optimization of glycemic control. The optimization of glycemic control in critical care trauma patients could reduce morbidity, and mortality in these patients [1-5]. This is also fundamentally true in cardiac surgery patients who were used as part of the modeling process [6-8].

CEGA was developed in 1987 for use in the quantification and assessment of patient's estimates of their blood glucose levels compared with the value obtained by a meter [12]. It was later used to compare the clinical accuracy of blood glucose levels generated by meters/monitors compared to reference, and it was utilized in this report because it has become the gold standard for blood glucose meter accuracy [13].

The utilization of this predictive model in real-time bedside monitoring for intelligent/directed therapy recommendation, guidance, and ultimately automation, will provide caregivers a means of enhancing patient safety and care. Furthermore, CGM identifies hypoglycemic and hyperglycemic excursions where conventional POC glucose values are not obtained, and results of this investigation demonstrate the utility of CGM in the critical care trauma setting. For the patients with an extended length of stay in the ICU, the results of this investigation substantiate the position that a patient specific neural network model (generated and trained with data from a single patient) may provide increased clinical performance and safety at the bedside. Further clinical trials will need to be pursued.

## Conclusions

Utilization of CGM in this patient population is clinically useful. Further investigation regarding the development and optimization of the real-time implementation of the neural network for prediction of glucose is ongoing and warranted based on this preliminary report.

## Consent

Written informed consent was obtained from the patient for publication of this report and any accompanying images. A copy of the written consent is available for review by the Editor-in-Chief.

## Competing interests

The authors declare that they have no competing interests.

## Authors' contributions

SMP and BDC conceived and wrote the mathematical algorithm. TJP, SMP, BDC, and RJB were involved in conception and design. JDL, DS, TS, TJP, MJB, SMP, and AC were involved in data acquisition. TJP and MJB cared for the patient. All authors were involved in interpretation of the data. TJP, MJB, SMP, AC, and BDC were involved in drafting the manuscript or revising it for critically important intellectual content. All authors have given final approval of the version to be published.
